# Self-Sovereignty Identity Management Model for Smart Healthcare System

**DOI:** 10.3390/s22134714

**Published:** 2022-06-22

**Authors:** Pinky Bai, Sushil Kumar, Geetika Aggarwal, Mufti Mahmud, Omprakash Kaiwartya, Jaime Lloret

**Affiliations:** 1 School of Computer and Systems Sciences, Jawaharlal Nehru University, New Delhi 110067, India; pinky82_scs@jnu.ac.in (P.B.); skdohare@mail.jnu.ac.in (S.K.); 2 Department of Engineering, Nottingham Trent University, Nottingham NG11 8NS, UK; geetika.aggarwal@ntu.ac.uk; 3 Department of Computer Science, Nottingham Trent University, Nottingham NG11 8NS, UK; mufti.mahmud@ntu.ac.uk; 4 Computing and Informatics Research Centre, Nottingham Trent University, Nottingham NG11 8NS, UK; 5 Medical Technologies Innovation Facility, Nottingham Trent University, Nottingham NG11 8NS, UK; 6 Department of Communications, Universitat Politècnica de València, 46022 Valencia, Spain; jlloret@dcom.upv.es

**Keywords:** internet of things, blockchain, self-sovereign identity, IoMT, security, privacy

## Abstract

An identity management system is essential in any organisation to provide quality services to each authenticated user. The smart healthcare system should use reliable identity management to ensure timely service to authorised users. Traditional healthcare uses a paper-based identity system which is converted into centralised identity management in a smart healthcare system. Centralised identity management has security issues such as denial of service attacks, single-point failure, information breaches of patients, and many privacy issues. Decentralisedidentity management can be a robust solution to these security and privacy issues. We proposed a Self-Sovereign identity management system for the smart healthcare system (SSI-SHS), which manages the identity of each stakeholder, including medical devices or sensors, in a decentralisedmanner in the Internet of Medical Things (IoMT) Environment. The proposed system gives the user complete control of their data at each point. Further, we analysed the proposed identity management system against Allen and Cameron’s identity management guidelines. We also present the performance analysis of SSI as compared to the state-of-the-art techniques.

## 1. Introduction

Blockchain plays a crucial role in healthcare applications, from improving medical record management, enhancing insurance claim processes, and accelerating clinical/biomedical research to advancing healthcare data by recording on the ledger. Blockchain technology can provide feasible and secure solutions to healthcare applications. The blockchain**’**s main characteristics, i.e., decentraliseddata management, data provenance, immutable audit trails, high availability, and, most importantly, security and privacy, increase the usability of blockchains in healthcare applications compared to traditional databases [1]. 

In smart healthcare applications, patients are implanted with wearable biosensors on their bodies and non-wearable sensors in nearby environments. These wearable biosensors and non-wearable sensors collect vital and biological data (e.g., cardiac activity, pulse rate, blood pressure, temperature, etc.). Biological data and personal patient profiles are addressed as an Electronics Health Record (EHR). The security and privacy requirements for an EHR have become more difficult and necessary as the movement to an EHR is one click away from being across the world. Challenges emerge as more health data is collected from wearable devices and Electronic Health Record (EHR) systems. The current centralized smart healthcare system has data isolation, data ownership, accountability, security, and privacy issues. Further, patients do not have control over their health data; the self-sovereignty concept is an excellent way to deal with these privacy issues. The current centralized concept is better for the scalability and mobility of the system. However, it is not good in terms of privacy, security, usability, single-point failure, and system complexity [2]. 

Identification is essential for public health management and quality delivery of health services to the end-user. Patients should be uniquely identified in the smart healthcare system to access the appropriate medical treatment and services. The service providers should also ensure that they provide consistent and correct services to the right person. The unique identification of patients helps researchers and administrators analyze records in order to generate statistics and other data planning, pandemic management, treatment improvements, tracking a patient in case of spreading diseases like covid, emergency response, and many more. Further, health insurance companies must also identify a patient to ensure the correct claims are submitted and provide insurance money based on the patient**’**s treatment history [3]. Smart healthcare consists of smart medical equipment, wearable sensors, or the internet of medical things, making identity management difficult for health service providers [4]. We can summarize that the healthcare system needs a secure, inclusive identity management system to provide quality health services.

The existing centralized identity management system for smart healthcare faces security, privacy, single point of failure, and interoperability issues. Further, individuals are given fewer or no options to control their health data and data transactions encompassing how, where, when, by whom, to whom, by what time, and which specific data is shared. The right of users to control and rectify personal information, including health information, has decreased in the digital era [4,5]. To solve the issues of a centralized identity model and patient privacy, the Self-Sovereign Identity Model (SSI) is an emerging concept of identity management. An SSI is a decentralised and owner-centric identity model that can solve the identification issues of a smart healthcare system. 

In this paper, we are proposing a decentralised identity management SSI for smart healthcare to provide patients control over their EHR. The proposed identity model covers the IoMT identification and gives control of device data to the device owner. The smart healthcare system has IoMT devices or sensors at different stages, like sensors installed with patients for remote monitoring, wearable devices, patient motion detection, and at hospitals to measure different health parameters. In the proposed identity model, the owner of the device (mostly the patient) has complete control of the sensors or the IoMT devices that collect data, and the owner chooses to share the information. 

The motivation of this research is to consider the IoMT device as an essential identity in the smart healthcare system. The identity management system aims to ensure that the service provider provides services to the trusted user based on the trusting relationship with an identity provider. However, there is no limit on the IoMT for providers to offer their services to any requestor. Traditional identity management systems focus only on real users**’** identities and negate the end-users like the application, the IoMT. Researchers were motivated to do this research to provide solutions to these limitations. 

The contribution of this paper can be summarized as follow:

- First, a system architecture is presented for a Self-Sovereign Identity Model for smart healthcare, including the IoMT network. The IoMT network is integrated with the smart healthcare distributed network.
- Second, the registration and authentication process of stakeholders in smart healthcare is presented along with the smart device installed or the patient’s collected EHR, registration, and authentication in the smart healthcare system.
- Third, we have implemented a prototype for the proposed SSI model using the permission blockchain, Hyperledger Indy, to collect the results for performance analysis.
- Finally, the proposed identity model is analysed with respect to the Allen identity model rules. Further performance analysis with respect to the execution time and storage is presented. The proposed distributed identity model gives complete control of personal data to the data owner. The patient and other stakeholders can choose the limited disclosure of personal information.

The organisationof the paper is as follows. Section 2 describes Preliminaries on Smart Healthcare and Identity Management concepts. In Section 3, we reviewed the existing identity model based on blockchains and without blockchains for smart healthcare. Section 4 describes the proposed identity model with architecture, communication flow, and process. The experimental implementation, results and their analyses are discussed in Section 5 and Section 6, respectively. Section 7 concludes the research paper and discusses future direction.

## 2. Preliminaries on Smart Healthcare and Identity Management 

### 2.1. IoT Enabled Smart Healthcare Model

The internet of things (IoT) has been used as a potential solution to reduce the pressure on the healthcare system and provide healthcare services to everyone, anytime and anyplace. A large amount of research focuses on this direction. It shows the considerable use of the IoT in healthcare, such as remote monitoring of specific conditions, aiding rehabilitation through constant monitoring of a patient’s progress, constant monitoring of patients using wearable devices, and many more. Baker et al. presented a range of uses for the IoT in healthcare and proposed a unique identity model for future IoT-based healthcare systems [5]. 

**Figure 1:**
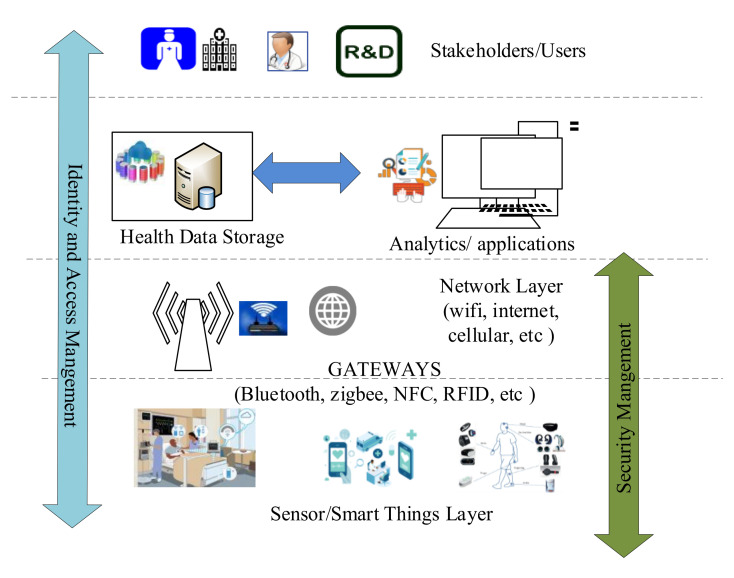
IoT enabled healthcare system.

Figure 1 presents the IoT-enabled healthcare system. Figure 1 captures all stakeholders of smart healthcare. The healthcare system comprises many stakeholders such as doctors, hospitals, clinics, pharmaceuticals, insurance companies, researchers, and core healthcare “patients.” The second important factor in the IoT-enabled healthcare is that the sensors are deployed with patients, and the generated large data is sent to the storage location. The storage server, like cloud, blockchain, or any database, stores this large health data, and the end-user applications access this data for further analysis and provide the services. Further, machine learning, deep learning, and soft computing or other computation techniques are used in the analysis to get specific results. 

In this work, we present the four key players as follows:

Healthcare Consumers: People or patients who receive healthcare services, treatment, or care. Patients access their healthcare records and share the same with doctors or hospitals, or other healthcare providers party to get the health services. 

Healthcare Regulators: Government institutes or public health departments that regulate health services among consumers. Health regulators monitors and fame the policies related to healthcare services. They aggregate the health data and process it to make new healthcare frameworks or policies. 

Healthcare Providers: Any entity that provides healthcare services to patients. It can be doctors, hospitals, nurses, ambulances, clinics, and others who are responsible for delivering health services. They collect the health data to provide health services. 

Industry Representative: Includes pharmaceutical firms, insurance companies, drug manufacturers, and medical device companies. They help operate the healthcare system and provide the latest and advanced solutions for health services. They collect the health data to provide good solutions to advance the health system. 

### 2.2. Digital Identity Management System

An identity management system (IDMS) or digital identity management system contains a set of rules and conditions for authentication, authorization, and the system’s access control. The IDMS ensures that only authorized entities can access the services in an organization. The core entities of any IDMS are the user, the identity issuer, and the service provider. In most IDMSs, a single centralized authority, like an organization, controls and owns the digital identities of specific organizations or systems [6]. 

Mainly, three types of IDMS models have been present since the internet’s beginning: Centralized Identity, Federated Identity, and user-centric Identity/decentralised identity model. The service provider authenticates users in the centralized Identity model before providing a service. Here, the service provider controls the identities and provides the credentials to access each service and time. The centralized system is the model we have been using for a long time: government ID, license card, college identity card, voter ID, Facebook, Twitter login, and so on. The identity issuer (mainly government and service provider) issues the identifiers and credentials to the user in the centralized identity model [7,8]. 

In the federated identity model, the identity provider manages the identities of more than one service provider. Users register for identity providers and can access the services from a federation. There are three popular federated identity protocols available: SAML, OAuth, and OpenID since 2005. Using protocols like OpenID Connect, social login buttons from Facebook, Google, Twitter, LinkedIn, etc., are now a standard feature on many consumer-facing websites [8,9].

In the user-centric identity model, the user controls its identifiers and defines a policy to share the attributes with the service provider to access the service. The decentralised identity model is based on peer-to-peer connection and does not have a centralized authority to manage the identity of a system. uPort, ShoCard, BitID, and soverin are examples of decentralised identity models. Further, the self-sovereign identity (SSI) model is a decentralised model that facilitates the recording and exchange of identity attributes and the propagation of trust among participating entities [9,10]. 

### 2.3. Self-Sovereign Identity (SSI)

In February 2012, a developer, Moxie Marlinspike, first wrote about the “Sovereign Source Authority” and mentioned that “individuals have a right to an “Identity”” [10]. Simultaneously, In March 2012, Patrick Deegan also started working on an open-source framework that gives the control of a digital identity to the user [11]. SSI introduces a layer of flexibility and security in distributed identity management systems. SSI is the concept where organizations and individuals have whole ownership of their identities along with self-defined attributes and identifiers, while the distributed identified management system (DIMS) uses the user’s already existing trusted credentials like PAN, Voter ID, Passport, etc [7]. 

In the SSI model, there is no central authority that holds user data and passes data on to other parties on a request. The user holds his/her own data. The cryptography and distributed ledger technology allow users to present claims about identity, and others can verify it with cryptographic certainty [11].

### 2.4. Architecture of Self Sovereign Identity Model

**Figure 2:**
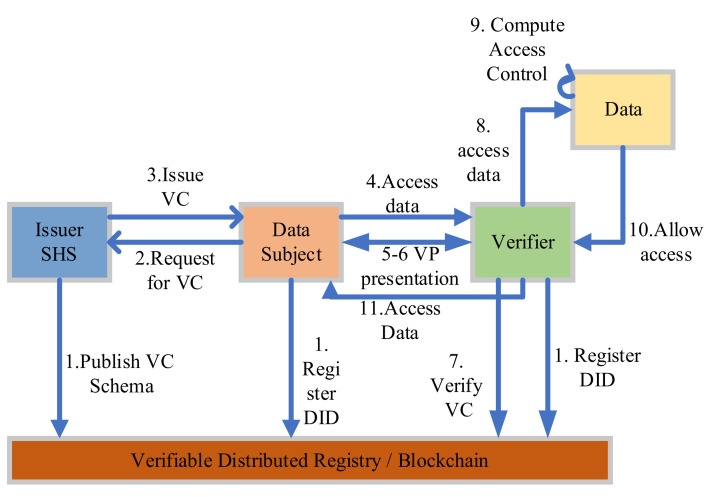
SSI communication Sequence.

The SSI model uses the core concept of identity management, blockchain or distributed ledger technology, distributed computing, and cryptography and provides a user-centric identity model. These concepts have been well established for a long time, and SSI put them together to create a more secure, persistent, and interoperable identity model. Figure 2 represents the sequence flow along with the important component of the SSI model. The conceptual architecture of the SSI model has four layers: Identifiers and Keys (DID), Secure communication and Interfaces, Verifiable Credentials, and Governance. Further, these four layers need seven building blocks to achieve the user-centric identity management goal [12,13]. The seven building blocks are:

- The trust triangle (issuer, holder, and verifier): Issuers are the source of credentials. The holder saves credentials issued by the issuers in its digital wallet and presents proof of claims when a verifier requests. The verifier verifies the credentials presented by the holder.
- Verifiable credentials or digital credentials: The digital equivalent of physical credentials are the verifiable credentials to prove the identity. The subject of the credentials creates a set of claims, and the verifiable credentials contain those claims. The issuer in the SSI model issues the verifiable credentials.
- Digital wallets: Digital wallets store credentials and other sensitive data and work with digital agents to securely exchange credentials among peers.
- Digital Agent: Digital agent is a software on the digital wallet that provides security to the digital wallet, participates in secure credentials exchange, and forms connections via a decentralised, secure message protocol. Edge Agents and cloud agents are two general categories of the digital agent.
- Decentralised Identifiers (DIDs): DIDs are decentralised, cryptographically verifiable, resolvable, and unique identifiers. DIDs are combinations of the private and public keys of a user. DIDs are decentralised by the nature that makes credentials available at all times for verifications. DIDs create a secure, unique, and private peer-to-peer connection between two parties who agree to connect with each other based on their requirements. The identity owner has complete control of the DIDs.
- Verifiable Data registries: A DID can be registered with any type of decentralised network, verifiable data registry, or even exchanged peer-to-peer. Blockchain can be a vital choice for verifiable data registry because a blockchain is a highly tamper-resistant transactional distributed database that no single party controls.
- Trust Framework: The trust framework contains the set of business, legal, and technical rules to use the SSI infrastructure and enables interoperable digital trust ecosystems of any size and scale.

The basic steps of information flow in the SSI model are:

- The issuer issues the verifiable credentials to the identity owner/holder. The VC includes the claims and attention.
- The user/holder stores this information himself. Users and holders can be the same sometimes. Furthermore, the VC holders have complete control of the VCs.
- When the user wants to access any service, he/she presents its VC to the verifier.
- The verifiers verify the VC without connecting the issuers. The verifier connects with a distributed registry (blockchain), verifies the user, and grants authorized services.
- The distributed verifiable registry has the VC schemas and DID, which helps in user verification.

The SSI model is an advancement of DIMS and user-centric identity. The main focus of the SSI model is that the user must be the controller of the identity, and the identity must come with interoperability across multiple locations with the user’s permission. There is vast literature available that writes about Self-Sovereign Identity Models. Here, we can summarize the ten principles of SSI. These principles focused on the user’s identity control, system transparency and fairness, and interoperability. Table 1 defines the ten principles of SSI and categorizes them based on focus area [13,14].

Alex Preukschat and Drummond Reed analysed and listed the major features and benefits of the SSI model. The SSI model can help in the following areas: fraud detection, reducing customer onboarding cost, auto authentication, auto authorization, automated workflow, data security, privacy, protection, portability, and much more [15].

Limitations:

This part addresses the challenges and limitations of developing the fully decentralised identity system or SSI system. The SSI is a distributed identity model and relies on the DID and Blockchain system, so blockchain performance directly affects the system. The issue faced in blockchain implementation, like storage limitations, scalability, predefined set of users, and so on, is directly accepted in the SSI model. Key storage is also an essential part of the implementation of SSI. When DID is created, the private keys and verifiable credentials are stored in secure storage with the user. The challenges come when the user loses the secure storage for any reason. In that case, it is like the user losing their access to identity.

## 3. Literature Survey

In this section, we have reviewed and analysed the literature on centralized and decentralised identity management systems. As the literature on SSI and distributed identity in smart healthcare is scarce, we consider access control and identity management in smart healthcare, e-health, and traditional healthcare. Some surveys are presented in the open literature on identity management based on blockchain technology. X, Zhu et al. presented the survey on blockchain-based identity solutions for the internet of things. The survey analysed the identity solution for IoT digital identity management and studied the recent increment in blockchain-based SSI solutions for IDMS [15]. Further, Kuperberg surveyed essential aspects of blockchain-based identity systems like compliance and liability, regulations, standards, integration, and user-friendliness [16].

We divide the literature analysis into a centralized identity model and a decentralised or SSI identity model for smart healthcare. 

### 3.1. Centralised Identity Model 

The health information system contains individuals’ personal information and critical health data. Bouras et al. stated that the current centralized identity management system is good in terms of scalability and mobility. However, the centralized identity model is poor regarding security, privacy, usability, single point of failure, and complex ecosystem [17]. 

Aghili et al. proposed lightweight authentication and an ownership transfer protocol (LACO), a secure and energy-efficient protocol that provides authentication and key agreement. The proposed work also covers the access control of health data and preserves doctor and patient privacy. The author designed a threat model for IoT and analysed the proposed protocol against around eleven security attacks. Further, the authors presented a comparison between the proposed model and the ZZTL [18] protocol [19].

Yang et al. proposed a big data storage system with self-adaptive access control to preserve privacy in smart IoT-based healthcare. The proposed work aims to provide emergency and normal access control and prevent duplication from saving space. Further, the system supports sharing encrypted medical files from IoT networks to different domains by applying the cross-domain sharing policy [20]. PASH, privacy-centered access control for the health system, is proposed. The proposed system revealed that only attribute names of access policies and attribute values are encrypted and stored in records because the attribute values have sensitive information, not the attribute names. Further, the security analysis shows that PASH is also secure as per the standard model [21]. Farid et al. present identity management solutions for IoT and cloud computing-based personal healthcare systems. The solution uses biometrics to perform the authentication in the system. However, the framework does not have user consent and is a combination of federated and centralized identity management. The proposed work does not present an end-to-end security analysis [22].

### 3.2. Decentralised Identity Model

As we have already discussed the properties and technical aspects of the Self-Sovereign Identity Model, the SSI model is a robust solution for protecting the data owner’s privacy as SSI gives its owner control of identity. Further, Houtan et al. analysed identity projects based on the SSI model, such as uPort, Soverin, evernym, ShoCard, TheKey, and other projects based on blockchain technology for the patient identity system [22]. 

Augot et al. developed a zero-knowledge proof-based solution for identity management. However, the proposed framework has two significant drawbacks. First, the authentication is not free. As the authentication is encoded with bitcoin transactions, the users have to pay a transaction fee to the miners. Second, the bitcoin transactions are public, so the user’s privacy is at risk [23]. Liang et al. presented personal health data management using blockchain and Intel SGX. The intel SGX stores the healthcare records; these records are trusted timestamping and free from redundancy, preserving both availability and accountability. However, the research does not include the identity management of the IoMT device [24]. 

AU et al. also proposed user-centric and privacy-preserving identity management for the distributed e-health system. Healthcare consumers maintain pseudonymous identifiers for use in different healthcare systems. However, this work does not include the implementation of the proposed architecture in an e-health system controlled environment and particle deployment in an e-health system [25]. Shuaib et al. explore the applicability of blockchain-based SSI solutions for healthcare, their advantages, and their requirements. Further, they proposed a model to demonstrate the use case of SSI. However, this work did not present the proposed model’s formal implementation and performance analysis [26]. Mikula et al. proposed identity and access management for EHR in the healthcare system to support authentication and authorization (use this in comparison). This paper does not cover IoMT device authentication and authorization [27]. Further, Zoho et al. applied sovereign identity claims to provide the distributed data vending system for the personal healthcare system [28]. Buzachis et al. also used uPORT, a self-sovereign identity platform, to identify patients in healthcare [29,30]. In recent times, researchers have included new technologies along with blockchain to secure the health system. Neelakandan et al. used deep learning with blockchain to secure the healthcare and diagnostic model [31]. Kamalraj et al. applied an interpretable filter-based convolutional neural network in the healthcare system for glucose prediction and further analysis [32]. Harshavardhan et al. proposed an optimization model for healthcare systems using LSGDM with biogeography-based optimization [33]. It is clear from the literature survey and the surveys mentioned earlier that no single distributed solution is fully distributed, covering users’ consent, privacy, and compliance with privacy standards [34,35]. The proposed research has the potential to be used in a smart city environment, such as smart traffic management [36,37,38]. Smart cities can be connected with smart healthcare to reduce the insurance claim process time and prevent fraud in the traffic-centric health insurance process [39,40,41,42]. 

## 4. Proposed Framework

We proposed an SSI model to manage the digital identities for a smart healthcare system. The proposed model includes the IoMT devices and provides digital identity to all stakeholders in smart healthcare. Further, the model gives complete control to the data owner at the time of sharing to PII and PHI. Whenever a user wants to access another user**’**s data, the requester**’**s data must authenticate himself to the requestee user. This provides the security and privacy of personal data and users**’** health data. We proposed a distributed blockchain-based SSI that does not require a central authority to control the identity lifecycle. The following section will explain the whole SSI model in detail. 

### 4.1. High-Level System Architecture of SSI Model for Smart Healthcare

**Figure 3:**
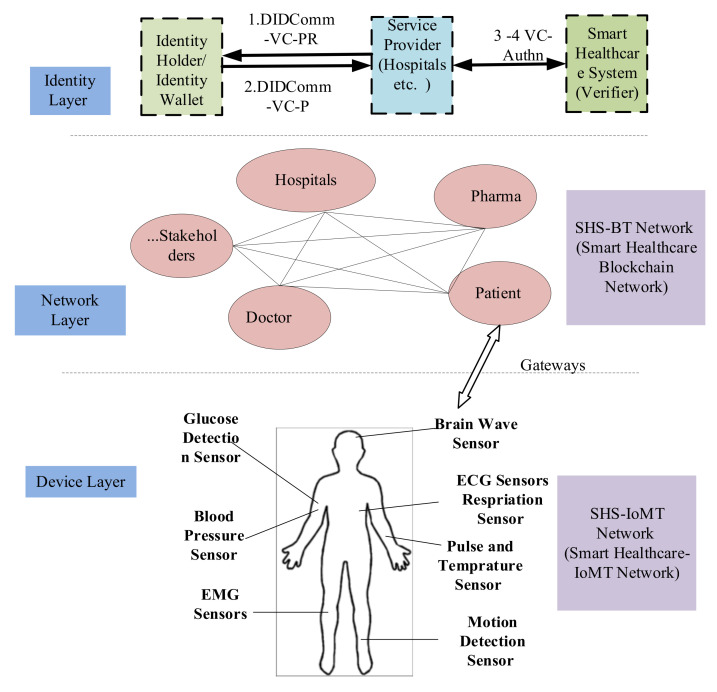
SSI-SHS architecture.

In this section, we present the high-level system architecture of the proposed model for the smart healthcare system. Figure 3 explains the main components of the SSI model of smart healthcare system (SSI-SHS) with reference to the SSI architecture presented in Section 2. The three main access-based roles of the SSI model are Subject (Identity Holder), Issuer (Identity issuer), and Verifier (Identity Verifier) in the context of smart healthcare as follows: the smart healthcare system (SHS) issues the verifiable credentials to all stakeholders (patients, doctors, labs, researchers, and others) based on DID. The subjects who hold the identity can be any stakeholders, and we include IoMT devices to cover the end-to-end information flow of smart healthcare. The verifier is the entity that provides any kind of service to others. For example, if a doctor wants to access the data of any medical device, in this case, a patient who owns the device verifies the doctor’s identity directly without any help from SHS (the issuer).

Further, all the stakeholders are in the same blockchain network (SHS-BT), and any entity that wants to access health services must register on the SHS-BT network. For the transactions, we will discuss the generation of transactions (identity management related) in the coming sections, stored at the SHS-BT blockchain distributed ledger (Li). The owners of IoMT devices register their devices on SHS-BT by providing the DID of devices along with their own DID. The SSI-SHS uses blockchain for verifiable data registry (VDR) also. 

VDR sets the rules in the distributed system for entities to create identifiers as per their own rules. VDR is a role or system that mediates the creation and verification of identifiers, verifiable credentials schema, keys, and other relevant data, such as public keys, revocation registries and so on, which are required in the verification of verifiable credentials. 

### 4.2. Communication Sequence Flow

In this section, we will describe the flow in SSI-SHS. The backbone of communication is DID communication between the Edge Agents of respective users. A user contains the user’s Edge Agent, front-end DID wallet, secure element, and micro ledger. The URL consists of a community resolver, a driver for DID methods, and a cache. We choose Sovrin [34] to demonstrate the interaction of DID among the users. A steward, a DID syntax checker, cache and resolution result constructor, and serialization validators are part of Sovrin. The VDR is any blockchain network. 

The system uses an agent that is a delegated entity by the DID subject. The agent controls the agent-to-agent DID communication, DID wallet cryptography-based operations, and sharing of credentials to authorized agents as per the relationships. Agents are categorized as Edge Agents and cloud agents. The Edge Agent resides within the wallet software locally. The cloud agent resides in the cloud and has extended features like identity wallet backup to the cloud, 24/7 DID communication when an Edge Agent is offline, data storage in the cloud, and key management. We use an Edge Agent (EA) in the proposed system. 

To explain the architecture, we take the most common communication in smart healthcare, where a patient wants to talk with a doctor, share health data, and device readings to get medical service. To establish the communication, the patient’s Edge Agent first queries the doctor’s DID from the Edge Agent to the community resolver within the universal resolver (UR). Then DID methods return the DDO of the doctor’s EA to the UR through VDR interaction. Now, the patient’s EA retrieves the DDO from UR. After that, the patient’s EA establishes the DID communication as per the data present in DDO. 

**Figure 4:**
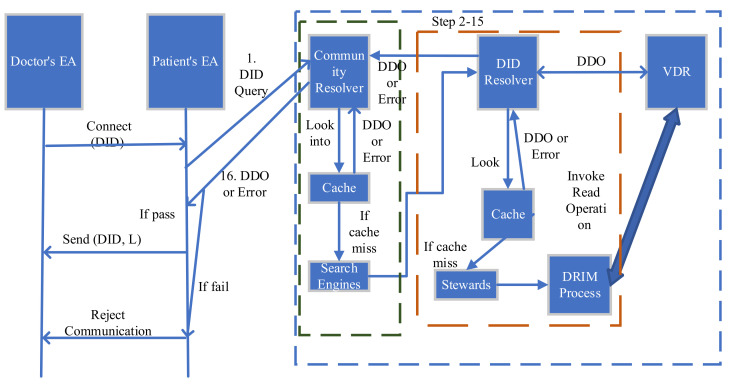
Authentication Process.

The whole smart healthcare identity architecture can be discussed in two parts: high-level user interaction named “SSI-SHS: SSI for smart healthcare system”; and the second part, “SSI-SHS-IoMT: Interaction of IoMT to SSI-SHS”, is a network among sensor and patient named as IoMT network. The term “IoMT” covers all types of medical devices, sensors, and other smart medical devices with the patient, as elaborated in Figure 4, the high-level SSI-SHS architecture.

*Part 1: SSI-SHS*

Phase 1: Registration: Identity Wallet and Agent Installation

In the registration phase, the shareholders create their own DID with the help of a digital wallet and Edge Agent. We described the registration of patients on the SHS-BT network. The following steps describe the process of getting the verifiable credentials from the SHS and registration:

Step 1: The patient installs the digital wallet software and initiates the creation of the first Edge Agent. The user uses the wallet to receive credentials from various entities and presents these credentials to prove himself on the system. 

Step 2: The Edge Agent (EA) creates a DID for agent communication, credentials for secure element and link secret (link secret is used in DID relationship establishment through a blinded commitment).

(1) $$EA_{patient}\to DID_{EA},Cred_{SE},linksecret$$

Step 3: The Edge Agent requests the Secret Element to create verifiable credentials (VC) and a VC presentation (VCP) for specific credentials schema (CS). The EA requests different types of keys like DID keys for signing and verification, Agent Policy (AP) keys, and encryption and decryption keys for the wallet.

(2) $$EA_{patient}\;Req\left(VC,VCP,CS\right)\to SE$$

Step 4: The Secret Element (SE) stores the following: VC, signing key of DID, decryption key of wallet, and AP keys.

(3) $$SE\leftarrow stores\;VC,PR_{patient},D.Key_{wallet},Key_{AP}$$

Step 5: The SE returns the following to the front-end wallet: VCP for CS, DID verification keys, and decryption keys for wallet backup.

(4) $$SE\;return\left(D.Key_{wallet},VCP,PR_{patient}\right)\to DigitalWallet_{frontend}$$

Step 6: The EA asks to store the following in the front-end wallet: agent IDs, CS registry address P, and a link secret. The address P denotes the storage location of CS in the public ledger. After that, CS establishes the authorization level for each agent based on each different credentials. The newly added agent stores VCP at address P in the PROVE section of CS.

(5) $$DigitalWallet_{frontend}\leftarrow store\left(EA_{patient}ID,linksecret,P_{AP}\right)$$

The result of the above process achieves the SSI and ensures that the privacy of the identity system is preserved via control and confidentiality. The system provides minimum controllable disclosure of the proof to achieve control of identity. The system stores the user credentials in the decentralised key management system identity wallet to satisfy confidentiality. 

Further, the EA proves the authorization using VCP without disclosing the secret value defined in Step 6. The CS Address Commitment (CSAC) can also be generated via the VCP with CS address to achieve herd privacy in the system. 

The DID keys are composed of a signing and verification key, as defined in steps 3 and 5. The signing key is based on the Edwards curve Digital Signature Algorithm using SHA-2 and Curve 25519 (ED25519). Next, when a new relationship is started, the DID and verification keys are shared with other parties. Lastly, CA is created for backup purposes.

Phase 2: Authentication using DID Method

After installing the agent and identity wallet, if the doctor wanted to access the patient’s data, the doctor would have to send a DID communication request to the patient. The EA of the patient gets the invitation for a DID connection from a remote doctor. The process flow is as follows:

(6) $$EA_{doctor}connect\left(DID_{doctor}\right)\to EA_{patient}$$

Step 1: The EA of the patient asks the query to the Community resolver (CR) of the Universal resolver (UR).

$$EA_{patient}query\left(DID_{doctor}\right)$$

Step 2: The CR checks the cache first after receiving the DID query. 

Step 3: The CR returns the stored DDO (DID Document) immediately if the DID query hits the cache. The DDO is passed with other metadata like DDO metadata, and DID resolution metadata.

(7) $$CR\;return\left(DDO,DID_{doctor}\right)\to EA_{patient}$$

If the cache miss in Step 3, the CR invokes the resolution process (RP). The CR first chooses the driver that is similar to the DID method, as received in DID. The proposed system uses the DID method as defined in the sovran for demonstration and implementation purposes.

$$CR\;invoke\left(RP,method_{DID}\right)$$

Step 4: The driver passes the DID and DID Resolution Input Metadata (DRIM) to RP to get the DID method. After getting the DID method, the DRIM is passed to the DDO.

Step 5: The steward hits the cache to check that the DDO is present in the cache for the provided DID.

Step 6: If the cache hits, the steward returns the DDO immediately. If the cache misses, the steward resolves the DID query. 

Step 7: the steward first checks the DID format; the input DID should be in a standard format. The steward throws an error on any syntax or semantics error. 

Step 8: The steward passes the DID method to invoke the read operation to VDR. If the DID has a public DID URL, then the DID URL has to be dereferenced to get more information regarding the specific resources to be targeted in the DDO. The DDO resources are identified with the help of the DID URL components like query, path, and fragments.

Step 9: In the first VDR operation, the DDO is returned to the steward after processing the read operation on VDR. 

Step 10: The serialization validator validates the DDO format as per defined in the DID. The serialization format can be JSON, JSONLD, and CBOR as per the DID core data model standard. If the serialization validator gives an error on a query, then the steward forwards the error to the requestor (driver from UR). 

Step 11: The requested DDO is sent to the steward and to the resolution constructor. The resolution result constructor makes the representation form of the DID resolution result. 

Step 12: The resolution result constructor sends the DDO to the serialization validator, and the cache updates with DDO. 

Step 13: The serialization validator sends this DDO to the steward. 

Step 14: The steward sends the DDO to the driver of UR. 

Step 15: The driver of UR returns the DDO to CR and caches updates with the DDO.

Step 16: The CR passes the DDO to the patient’s EA.

(8) $$CRsend\left(DDO,DID_{doctor}\right)\to EA_{patient}$$

Step 17: After verifying the DDO, if the patient is satisfied and ready to connect, then the EA of the patient sends a DID communication message along with a delta of micro ledger.

(9) $$EA_{patient}\;send\left(DID_{commn},encrypt_{PR_{EA}}\left(\nabla L\right)\right)$$

The EAs use a message-based protocol to communicate and exchange a series of messages with each other. The delta of the micro ledger consists of the record of DID events from each EA. The delta represents the updates of the micro ledger, and it is organized in the Merkle tree. The EA exchanges the delta via authenticated encryption using the verification key of the EA. 

Step 18: The doctor’s EA stores the DID events in the relationship between patient and doctor. The micro ledger of the doctor stores the delta of the micro ledger sent by the patient to make sure that the patient and doctor have the same copy of the DID events. 

Step 19: The change of state of the doctor’s micro ledger is sent in response to the recording operation. 

Step 20: The doctor’s EA sends a reply of DID communication message and a copy of the micro ledger delta of the doctor to the EA of the patient. 

Step 21: The EA of the patient stores the DID events in the micro ledger and stores the delta of the micro ledger sent by the doctor. 

Step 22: The micro ledger sends a state change response to the EA of the patient. 

Step 23: The EA of the patient checks that the DID events are in sync with the doctor’s DID events. 

Step 24: The identity ledger (Li) stores the transaction as either failed or passed. The blockchain transactions include the EA of the requester and provider along with the status (pass or fail) and a hash of the ledger with a timestamp as below. 

Tx (EApatient, EAdoctor, status, timestamp, hash(L))

Figure 4 describes the authentication process of a doctor by a patient without any centralized system or hospital authority. These two phrases describe the registration and authentication of high-end users and stakeholders. The privacy and secrecy concerns in smart healthcare increase with the number of IoT devices in the system, and smart healthcare is loaded with lots of medical devices, and these devices are exposed and vulnerable to the outside world. 

*Part 2 SSI-SHS-IoMT: Interaction of IoMT to Healthcare SSI*

Here, we present the second part, where a medical device connected with a patient registers itself and authenticates in a smart healthcare system. We assume that all the medical devices/sensors (IoMT) are connected to the internet and pass the information to a remote doctor/hospital to analyze the patient**’**s health and provide health services. Now the patient owns his DID and VC and has some medical sensors and devices which send data to smart healthcare.

We design smart contracts for authentication named “DIDMaster**”** that provides the information related to the device after supplying the DID: (diddev.H(docdev). URIdocdev .stadiddev). The “AccessData” smart contract provides access to the data of the patient’s devices. These smart contracts are deployed on VDR, and the node in the SHS-BT network calls the smart contract by passing the correct parameter, as explained in the next part.

Phase 1: Registration of IoMT devices

Here, we consider that all the devices are bootstrapped in the patient’s environment. Now, the device registration of the device is with the patient. The patient is an onsite registration authority for devices in SSI healthcare. In smart healthcare, a device is owned by a single patient, and a single patient can own many devices. After receiving the VC from smart healthcare, the patients register their IoMT device and bind the ownership of the device. 

The Bootstrapping process:

1. Once the device is active, the patients send their own DID to the device.
2. The device creates an authentication token that includes the patient**’**s DID, signs this token with a private key (AuthToken1), and sends the token back to the patient.
3. The client creates another token (AuthToken2), including AuthToken1, and signs this with its own private key.
4. The patient calls the “DIDRegister” smart contract as a message sender and passes the device address. “DIDRegister” registers the assignment between the device and registrar with a tentative state.
5. The patient submits the AuthToken2 to a smart health system node which is a server application connected to the blockchain.
6. The SHS checks the validity of AuthToken2 and the registration status of “DIDRegister” (step 4). If both are valid, the identity provider node proofs the assignment of “DIDRegister”. Afterward, the “DIDRegister” changes the state to active.
7. The SHS node generates an AuthToken3 with a confirmation about the assignment, signs it with its private key, and sends it to the patient.
8. The client forwards AuthToken3 to the device.
9. The device verifies the signature of the SHS node with its built-in list (in a secured environment) and, if ok, adds the patient to its trust list.

Phase 2: Authentication of IoMT on network

Here, the patient who is the owner of the medical device has complete control over the data collected by their medical/IoT device. A detailed description of how the doctor accesses the data from IoMT with user consent:

Step 1: The patient provides the DID of a device to the doctor to access the data; after receiving the DID of the device, the doctor calls the “DIDMaster” smart contract bypassing the DID of the device and the patient. 

Step 2: The smart contract returns the details of the device: hash of the DID device, hash of DDO of the device, status of the device, and URI of the device. 

Step 3: If the status of the device is active, the doctor sends the DID to the verifier to verify the DID. 

Step 4: The EA of the doctor gets the address of “AccessData” from step 3.

Step 5: The EA of the doctor calls “AcesssData” by passing his own parameters: didoc, reqdoc; where diddoc is the doctor’s DID and reqdoc denotes the data set that doctor would like to access. Moreover, the smart contract AcesssData also checks whether the data access request reqdoc is valid. After the successful execution of AcesssData, a data request event, which contains the doctor’s request parameters, is emitted on the blockchain.

Step 6: Successful execution of the “AcesssData” smart contract saves on the blockchain. 

Step 7: The EA of the patient, on receiving a new data request, sends the DID of the doctor to the UR to resolve. Moreover, the further process is the same as “Phase 2: Authentication using DID Method”. 

Step 8: If the doctor**’**s data request passes all the validations, the patient**’**s EA invokes the smart contract AccessData with parameters diddoc, reqdoc, and Sigskpatient (Addrs doc. Reqdoc), which grants the doctor’s data access request, and saves the data access granted txn on the identity ledger (Li).

Tx (EA_patient_, DID_dev_, EA_doctor_, status_dev_, timestamp, hash(L)) (10)

Step 9: When a doctor**’**s data access request is granted, he obtains the access token Sig𝑠𝑘patient (𝑎𝑑𝑑𝑟doc, reqdoc) from the saved txn event and requests to download data from the service endpoint specified in the docdev. 

Step 10: To identify that the data requester is a doctor, the patient sends a random challenge 𝑟 to the doctor. Then, the doctor generates a tuple ⟨𝑎𝑑𝑑𝑟dctr, reqdctr, 𝑝𝑘dctr, Sig𝑠𝑘patient (𝑎𝑑𝑑𝑟dctr, reqdctr), Sig𝑠𝑘dctr (𝑟, 𝑝𝑘dctr)⟩ as the response. 

Step 11: The patient first checks that the access token Sig𝑠𝑘patient ( 𝑎𝑑𝑑𝑟dctr, reqdctr) is valid, which indicates patient authorization for the doctor making the reqdctr query on data collected by the patient’s IoT device. 

Step 12: The patient then checks that 𝑎𝑑𝑑𝑟dctr is derived from 𝑝𝑘dctr. Finally, it verifies the signature Sig𝑠𝑘dctr(𝑟, 𝑝𝑘dctr) to confirm that the response actually comes from the doctor. 

Step 13: If the doctor**’**s response passes all the above validations, the patient generates a download link and sends it back to the doctor. Otherwise, the data retrieval request is rejected.

**Figure 5:**
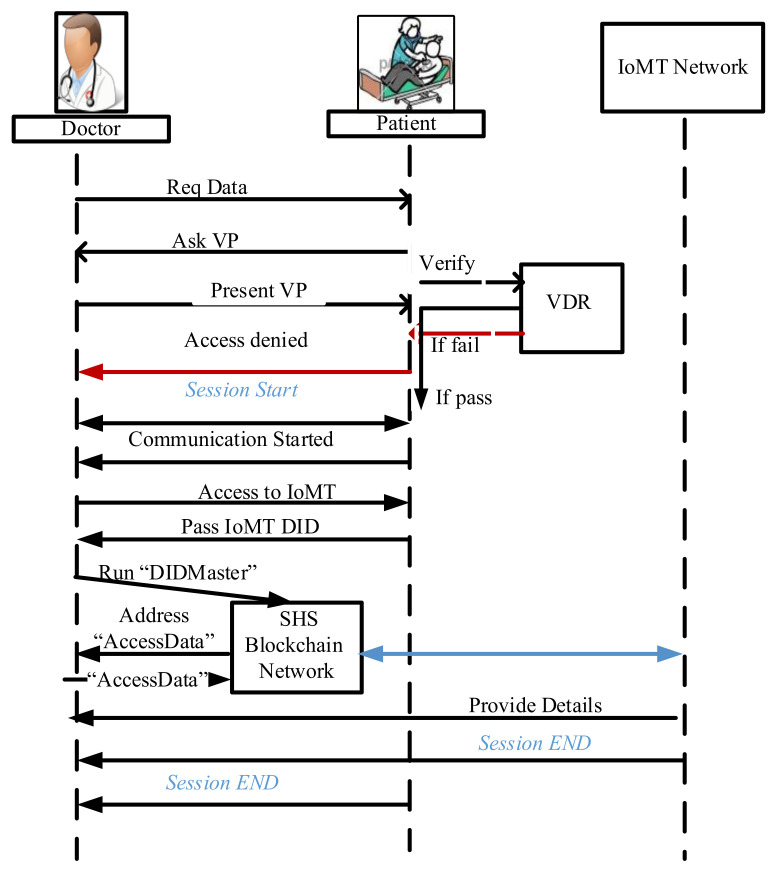
SSI-SHS process flow scenario: The doctor access the IoMT data.

We have described the registration and authentication process of all entities of smart healthcare in the proposed identity model SSI-SHS. Figure 5 presents the complete process of SSI-SHS; the most common scenario starts with a doctor who wants to connect a patient to a doctor who gets access to the IoMT data based on the request. Other communication also follows the same process. Any nodes who want to participate in smart healthcare first get the identity and then present the identity proof to get the healthcare services.

## 5. Implementation

A prototype of the proposed SSI-SHS identity model is implemented. The blockchain network (SHS-BT) is designed on Hyperledger Aries blockchain, a private network using four nodes: doctor, patient, hospital, and laboratory. Further, the patient’s IoMT device is a health band that measures the patient’s heartbeat, BP, and sugar. 

Terminology:

Identity Ledger (Li): The ledger is the verifiable directory that stores identity records and transactions. The public data like public keys, service endpoints, credentials schema, credentials definition, etc. define the identity record. The relationship between identity record and DID is 1:1, meaning each record has one DID. The DID is unique and resolvable via an identity ledger without needing any third-party centralized authority. 

Trust Anchor: A trust anchor (person or organization) that the ledger already knows, bootstrap others. In the smart healthcare system, we can think that an organisation(SHS), hospital, doctors, and other stakeholders must trust anchor roles that bootstrap other entities into the process. 

DID Creation: The DID creation is defined in the DID method, and according to the DID method, the node passes the minimum information for DID creation. The W3C organisationmaintains the DID specification registries that include all implemented DID specifications. 

Here, we took the minimum input parameter and generated the DID using the “genrateDID” function. The IoMT device generates cryptographically verifiable public and private keys in a trusted execution environment. The device stores the Private key in its secure environment and passes the public key to generate the DID.

$$genrateDID(“0x5576E95935566Ebd2637D9171E4C92e60543fg10”,\;\;“8806157fdcbcae265667576fa72d88568db7f9ca8b36tydfe3755ae80457eaf5”,\;\;“user:password@tcp\left(example\_connection\_string:3106\right)/”)$$

This “genrateDID” function returns the DID for the subject in a format:

did:abc: H3C2AVvLMv6gmMNam3uVAjZpfkcJCwDwnZn6z3wXmqPV 

Here, “*did*” is scheme, “*abc*” is DID method, and the remaining part is method specific identifier. 

DID Documents (DDO): The DID document, as per the W3C following parameter, must be present in DDO while creating a new DID for a node. The DDO expresses the cryptographical equations, verification methods, services, and controls. The services enable secure and trusted interaction of DID subjects with others. The verification method defines the verification of the DID subject by the verifiers. The DID resolver resolves the DID into DDO. We used hyperledger aries blockchain to implement the prototype. Listing 1 presents the DID and DDO used in a prototype implementation. 

**Listing 1:** Example of blockchain based security implementation DDO.

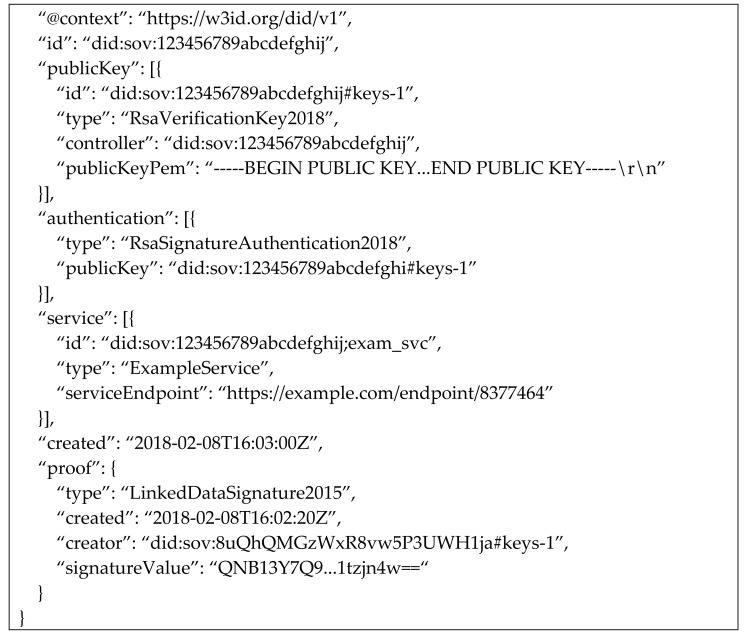



Verifiable credentials: Verifiable credentials represent statements made by an issuer in a tamper-evident and privacy-respecting manner. When an organisationissues verifiable credentials, they attach their public DID to the credential, and the verifier can verify the same without contacting the issuing authority. The verification method is presented in the DID document along with other attributes. The issuer cryptographically signs the VC. The VC includes proofs and claims for the subject. The sample of VC from the implementation environment is presented by Listing 2. 

**Listing 2:** Example of blockchain based security implementation credential schema.

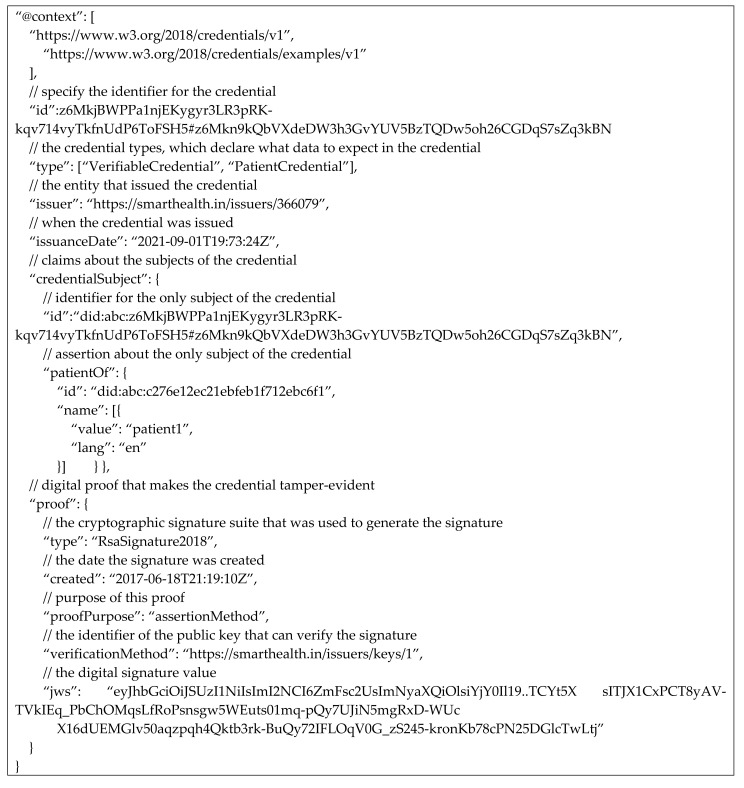



The Credential Schema is a document that is used to guarantee the structure and, by extension, the semantics of the set of claims comprising a Verifiable Credential. A shared Credential Schema allows all parties to reference data in a known way.

The ledger stores a number of different types of transactions. The transactions that:

- Write a new DID and DDO to the ledger.
- Update existing DDO such as rotating keys
- Define a new Schema name, version, and list of attributes for new credentials
- Define a revocation registry for specific credentials
- Update the revocation registry when the issuer issues or revokes the credentials.

Write the public key from a generated pair of signature algorithms for a specific credentials schema. 

## 6. Result and Analysis

### 6.1. Identity Framework Evaluation and Result Analysis

The SSI Identity Model for smart healthcare, including IoMT, has been described in detail in the previous section. Here, the SSI-SHS framework is evaluated and analyses the results derived from the prototype experiment. 

#### SSI-SHS Identity Model Evaluation

There are no standard criteria available on how to evaluate an SSI system. Allen proposed the SSI requirements focusing on personal data control, security, and privacy [12]. In the same direction, Cameron also presented “Seven Laws of Identity” [14]. This is a well-established framework for the digital identity system. NIST has a standard for “digital identity.” We reference both guidelines to evaluate the SSI-SSH. We have modified and deleted some rules per the framework requirement and the practicality of implementing the guidelines. For example, the interoperability of identities is designed within the smart healthcare system, but the interoperability outside the smart healthcare system needs a standardized format and procedure. 

The requirements and guidelines are divided into three groups: User control, Security and Privacy, and Portability. 

User Control: This group includes “Existence”, “Control”, “Consent”, and “protection”.

1. Existence: NIST defines that every digital identity must have a non-digital existence that manages and represents the online identity. In the proposed architecture, the device and the stakeholder generate their public and private key pair and register themselves on smart healthcare. The main focus is on the patient and his own devices.
2. Control: “Control” of the owner on their identity is proposed by Allen and Cameron. This principle defines that users must have control over their identity and be able to decide which part of their identity they want to share. They should be able to decide which data they share with others, for how long, and be able to refer to, update or hide the identity. In the proposed framework, multiple DID can be derived with a single key pair with different DDOs.
3. Consent: The use of the user’s identity should always be with the user’s agreement. The user should decide which information and with whom it is shared. Further, the user should decide the time; this also means what time the other party can have access to this information.

***Security and Privacy:*** This group includes “Access,” “Transparency,” and “Minimization.”

1. Protection: To preserve the freedom of the user and to keep the balance in the system, a censorship-resistant, independent, and force-resilient algorithm needs to be run in a decentralised manner.
2. Minimization: This law describes that the closure of credentials should be as minimal as possible. The minimum disclosure protects the privacy of the user. The proposed framework uses zero-knowledge proofs (ZKP) as verifiable credentials presentation. The ZKP allows cryptographically proven claims without sharing the actual information. The claims and proofs are present on the identity ledger, where a verifier can verify the specific claims.
3. Persistence: The lifetime of the digital address of identity/identifiers should be under the user’s control. The identifier should exist till the user wants it. In the proposed system, the revocation of the DID is covered, which fulfils this requirement.
4. One further principle could be privacy-preserving. Even though this is already partly integrated without explicitly saying it, the privacy-preserving design of services plays a key role in Self-Sovereign Identity. Reselling user-related information is a large business on the internet.

***Portability***

1. Access: Access to the user’s identity should be accessible to the user at any time. No intermediaries should prevent the user from accessing their identity. The distribution and access of data or identity should be accessible to the authorized parties only.
  In the proposed framework, only public information is available on the ledger, and the stakeholders have their personal information (or PII) on local storage. An Access Control List (ACL) is also designed on blockchain to prevent unauthorized access. If any party (doctor, hospital, pharmaceuticals, and other stakeholders) wants to access others’ information, they must first authenticate themselves in the system.
2. Transparency: The identity system must be transparent to each stakeholder. This leads to high trust and continuous improvement. Further, the participants can control the actions of each other and prevent and detect malicious actions from happening.
  The proposed framework is designed on a blockchain distributed network. Blockchain is the solution for transparency and trust.
3. Interoperability: The identities should be usable for many services; they should not be limited to a single service.

We proposed SSI for smart healthcare, and smart healthcare has many services with a large number of stakeholders. The identity information should be accessible by services in a standardized way. This should be created.

### 6.2. Security Analysis of SSI-SHS

In the proposed solution, private information does not store on blockchain. The PII is stored within the wallet (mobile wallet in implantation) in encrypted mode, and the wallet is secured using a fingerprint (or PIN or biometric feature the vendor provides). The proposed system defines the requirement of a secure interface while accessing the wallet and considers that vendors provide their implementation, so the security analysis of these implementations is generally challenging. Here, we suppose the vendor provides a secure way to protect the wallet and its data. 

Even though the data in rest compiled the privacy of the design concept, at some point, the private information must be processed by the service provider’s server. At the time of personal information processing, data privacy depends on the trust level of implementation and the segregation of the component. This implies that the highest level of privacy may only be achieved if a separate organisationoperates each component and there is a process set up to ensure the implementation does not store data that are supposed to be transient. Both of these may be done by regular organizational audits.

The security analysis of the issuance and authentication process can tell the security strength of any system. In the proposed framework, the issuance of the entity’s credentials and authentication is crucial. The analysis will be done for the security strength of both processes.

#### 6.2.1. Issuance Process Security Analysis

The issuance of VCs to the user consists of many steps, starting from getting the DID from the user and passing the VC to the user storing these VCs in a secure wallet. The identity requestor/holder and issuer communicate via a secure communication channel. The following threats can happen while issuing the credentials:

- The attacker gets the exchanged data between the issuer and holder;
- Man in the middle attacks over DID communication;
- Key Exposure attack;
- DDO forgery attack.

In the proposed SSI-SHS framework, the communication between the issuer and the requestor is protected at several levels. The certificate pinning is where the public key is associated with its host and is recommended and used in a prototype implementation. The framework considers the exchange of critical encrypted data and is implemented in the prototype. The certificate pinning prevents the man-in-middle attacks. 

Most of the attacks are rooted in key vulnerability exposure attacks. In the proposed system, the keys, VC, and secret links are secured elements (TEE). Moreover, the keys are maintained through DKMS. This method can prevent key exposure. 

Further, the wallet stores all data in encrypted form. For the DDO forgery, the attacker needs a trapdoor key with the help of the key exposure technique. Moreover, we have already discussed that the proposed framework is resistant to key exposure, so DDO forgery can also be prevented using the proposed framework.

#### 6.2.2. Authentication Process Security Analysis:

The authentication process can have the following attacks:

- Wallet attacks;
- Man in the middle attacks on DID communication;
- By passing an authentication attack.

All wallets at the user’s end need strong encryption. The encryption algorithm must be strong and searchable, and it should not depend on the storage technology of data in the wallet. The encryption technique should be able to hide the data pattern in the encrypted data and rotate the key to protect the wallet without having to re-encrypt the whole data. This would not be possible with the help of a trivial encryption algorithm. Some strong encryption, like Ethereum, uses AES-128-CTR cipher with decrypt and MAC, and Indy wallet uses HMAC-SHA256 and other identity wallets implemented while keeping the above two requirements. We used a sovrin wallet to store the keys and PII of the user in a prototype implementation. 

Two situations may threaten user personal information: the data are disclosed to the attacker during the authentication process, or the VC discloses more that the user consented to share. A common strategy mitigates the first threat by the solution: mandatory TLS usage with optional certificate pinning with additional application-level encryption. The second threat relies on Mobile Wallet implementation: it must ensure no data leaves the Mobile Wallet without the user’s consent. The proposed framework considers that each function call to obtain data has an exact authorization procedure associated, and the authorization is applied to the whole identity and each item separately: the user enables access to his identity, but to access personal information, an additional step is needed. The verification processes differ for each VC technology. During the verification process of basic PKI credentials, personal information is exchanged as attributes linked to the VC after VC verification. During verification, data are exchanged. From this point of view, the verification approach is more privacy-friendly and provides a much better solution.

### 6.3. Performance Analysis:

#### Execution Time Analysis

We first run the primary operation of the SSI-SHS network, i.e., registration of new stakeholders, registration of IoMT devices with a patient, authentication of an entity on the network, credentials issuance, and credentials verification. We recorded the execution time of these primary operations to evaluate the performance of the proposed identity model. We analysed the model in terms of execution time with 50 peers and ten medical devices. We put data on medical devices directly (not real-time monitoring). The execution time depends on many factors like connectivity, hardware, and program complexity, so the execution time varies from network to network. Here, we captured the data as our local machine.

**Figure 6:**
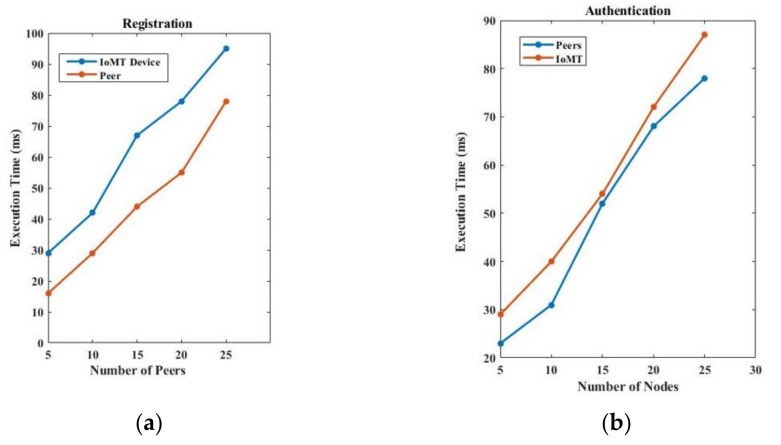
(**a**) Registration and (**b**) authentication time of stakeholders and IoMT devices.

Figure 6a presents the time taken in the registration of a stakeholder and the medical device of a patient. It is clear from Figure 6a,b that IoMT device registration and authentication take more time than the registration and authentication of stakeholders. The reason is that the IoMT device lacks communication power and energy. Furthermore, device registration and authentication include smart contracts, unlike the direct registration and authentication of stakeholders. Figure 6b presents the authentication time for stakeholders and medical devices. 

**Figure 7:**
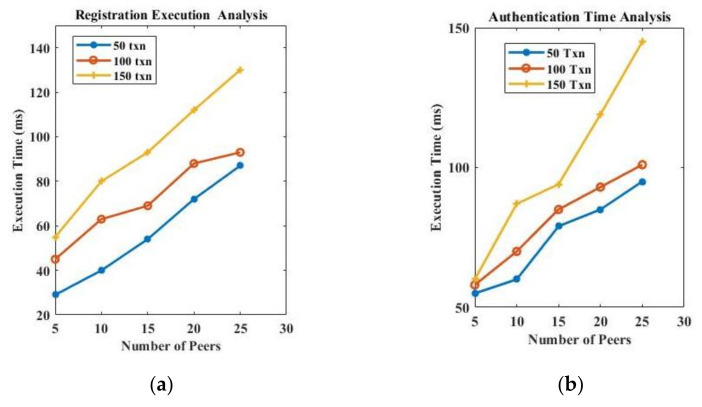
(**a**) Registration time on network scale; (**b**) authentication time on network scale.

Further, we recorded the registration and authentication time with a varying number of transactions (50, 100, 150 txn) in the network and drew it into Figure 7a,b. Figure 7a presents the registration time when the number of transactions increases on a network scale, and Figure 7b presents the authentication time when the number of transactions increases on a network scale. The scalability of the network depends on the infrastructure resources used in the implementation. However, as the number of participants increases in the network, the number of transactions used in both the registration and authentication process increases. If we use high computation and large storage sources, then increased transactions can be handled in a timely manner.

**Figure 8:**
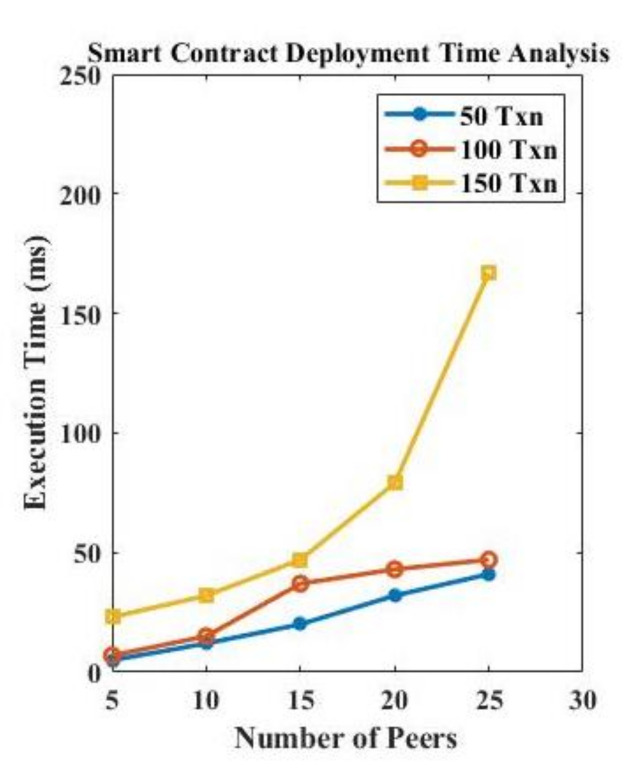
Contract deployment analysis.

**Figure 9:**
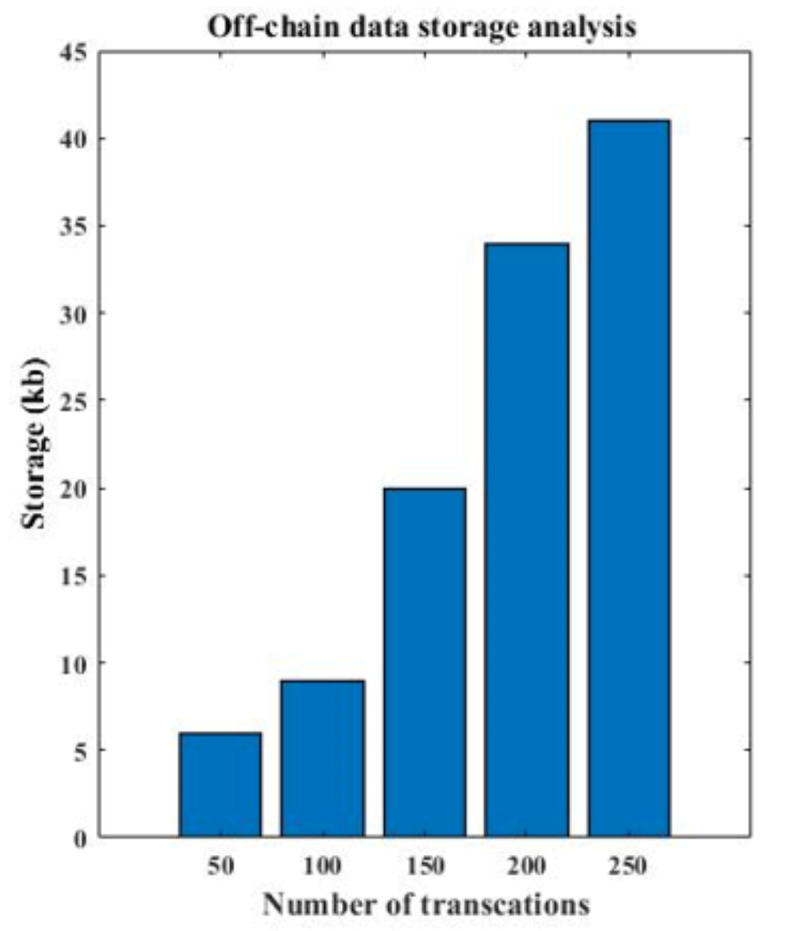
Execution time analysis of off-chain storage.

Figure 8 presents the time analysis and the contract deployment analysis for varying numbers of transactions and peers, and Figure 9 presents the execution time analysis of off-chain storage. The smart contract is deployed on the blockchain, and nodes trigger queries through smart contracts for different functionalities. As the network increased, the smart contract queries also increased. As shown in Figure 8, the execution time of a smart contract is increased with the number of nodes participating in the network. Further, the transactions storage also increases with the number of nodes in the network, as presented in Figure 9.

**Figure 10:**
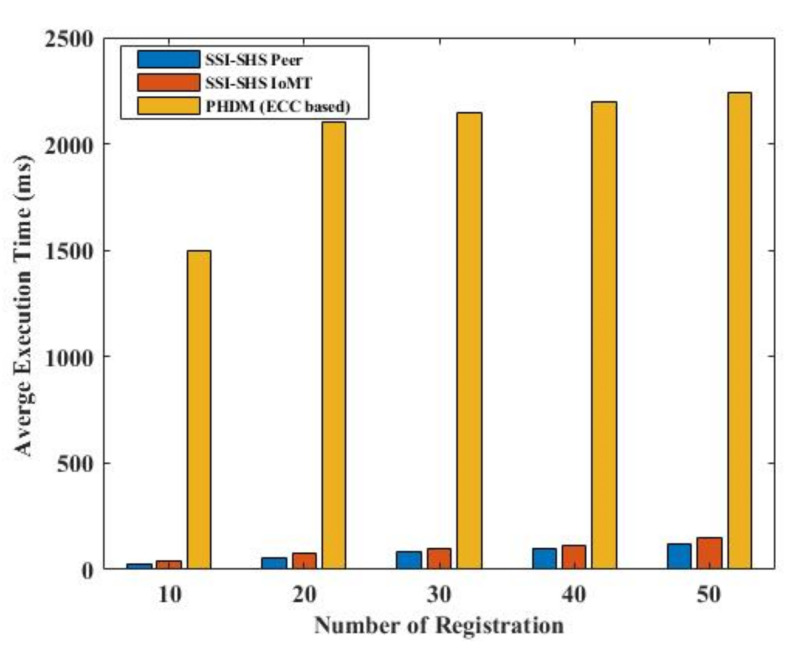
Performance comparison.

We compare our proposed identity model performance with the existing model proposed by Xueping Liang et al. [24] and Rafael Belchior et al. [30]. Our SSI-SHS provides better performance, as presented in Figure 10. SSI-SHS is better in both phases, i.e., registration and authentication. Further, we also include the authentication and registration of IoMT devices, which makes our approach much more robust to security issues.

## 7. Conclusions

The SSI system is proposed for smart healthcare (SSI-SHS) to protect the user’s privacy and give the owner control of health data. Smart contracts are designed to request the user’s data and provide IoMT data access to other trusted parties with a time limit. First, we designed a distributed network of all stakeholders of smart healthcare on a permissioned blockchain to limit the participants at the healthcare level, which provides application-level security. In the proposed identity system, the SSI-SHS and IoMT-SSI are connected through the common participants in both networks, like patients. The IoMT-SSI manages the identity of IoMT devices through the device owner rather than the device’s manufacturer. The device owner controls their health data even if the data is gathered via some smart medical devices. Further, the results and analysis show that SSI-SHS complies with the identity guidelines proposed by Allen and Cameron. For future research, the identity model can be expanded to make it interoperable with other smart parts of a smart city. For example, smart traffic management in smart cities can be connected with smart healthcare to reduce the insurance claim process time and prevent fraud in the health insurance process.

## Figures and Tables

**Table 1:** Principle of SSI.

User’s Control	Security	Portability
Users must have control of their data like which information can be seen the other	Keep identity information secure	Users can move anywhere without being tied to a provider
Existence	Protection	Access
Control	Persistence	Transparency
Consent	Minimization	Interoperability
Persistence		

## Data Availability

Research data will be available on individual requests to the corresponding author considering collaboration possibilities with the researcher or research team and with restrictions that the data will be used only for further research in the related literature progress.
